# Prevalence, outcomes and associated factors of SARS-CoV-2 infection in psoriasis patients of Southwest China: a cross-sectional survey

**DOI:** 10.1038/s41598-024-54424-y

**Published:** 2024-03-15

**Authors:** Yang Zou, Jing Xu, Ai-Jun Chen, Kun Huang, Shou-Min Zhu, Jian-Jun Li, Jin He, Jun-Zhi Li, Jian-Xia Xiong, Yu-Kun Fan, Chuan Liu, Yun Pan, Ping Wang

**Affiliations:** 1 Department of Dermatology, The First Affiliated Hospital of Chongqing Medical University, Chongqing, China; 2 Department of Dermatology, People’s Hospital Affiliated of Chongqing Three Gorges Medical College, Chongqing, China; 3 Department of Dermatology, Chongqing University Qianjiang Hospital, Chongqing, China; 4 Department of Dermatology, The People’s Hospital of Kaizhou District, Chongqing, China; 5 Department of Dermatology, The Third Affiliated Hospital of Chongqing Medical University, Chongqing, China

**Keywords:** Infectious diseases, Skin diseases, Public health

## Abstract

In this study we aimed to investigate the prevalence of SARS-CoV-2 infection in psoriasis patients, and outcomes of SARS-CoV-2 infection and associated risk factors. A cross-sectional survey was conducted from February 2023 to March 2023. Information was obtained with online questionnaire about psoriasis patients on demographic characteristics, clinical characteristics, SARS-CoV-2 infection and outcomes, vaccination, and routine protection against COVID-19. Logistic regression analysis was used to explore risk factors with SARS-CoV-2 infection and exacerbation of psoriasis. A total of 613 participants were recruited. 516 (84.2%) were infected, and associated factors were sex, working status, routine protection against COVID-19, COVID-19 vaccination, impaired nail, infection exacerbate psoriasis, and severity of psoriasis. Among the patients infected with SARS-CoV-2, 30 (5.8%) required hospitalization, 122 (23.6%) had psoriasis exacerbation due to SARS-CoV-2 infection, and associated factors were subtype of psoriasis, discontinuation of psoriasis treatment during SARS-CoV-2 infection, response following COVID-19 vaccination, and severity of psoriasis. Booster dose vaccination contributed a low probability of COVID-19 sequelae. COVID-19 vaccine’s effectiveness was unsatisfactory, while booster dose vaccination reduced the occurrence of COVID-19 sequelae in psoriasis patients of Southwest China. Patients treated with psoriasis shown to be safe, without a higher incidence of SARS-CoV-2 infection or COVID-19hospitalization compared to untreated patients. Stopping treatment during SARS-CoV-2 infection led to psoriasis exacerbation, so psoriasis treatment could be continued except severe adverse reaction.

## Introduction

Coronavirus disease 2019 (COVID-19), caused by severe acute respiratory syndrome coronavirus 2 (SARS-CoV-2) infection^1^, threatened the lives and health of people worldwide. Chinese government took measures to quickly control the SARS-CoV-2 epidemic which started in late 2019, then adopted the dynamic zero-COVID policy to prevent another nationwide COVID-19 outbreak^2^. Due to the high transmissibility of the Omicron variant and symptoms caused by Omicron variant were mostly mild and asymptomatic^3^, full-dose of COVID-19 vaccination rate in Chinese population was close to 90%, so on December 7, 2022, Chinese government announced the end of the dynamic zero-COVID policy and no more infected patients would be with mandatory isolation control^4^. Subsequently, Chinese population was subjected to a massive wave of SARS-CoV-2 infection impact, with more than 80% of the population infected^5^. Despite this, no studies have been conducted to determine the status of SARS-CoV-2 infection in Chinese patients with psoriasis.

Psoriasis is a chronic, immune-mediated inflammatory skin disease with systemic implication induced by a combination of genetic and environmental factors^6^. Psoriasis patients who received long-term therapy including biologics and systemic immunosuppressive medication treatment have an increased risk of infection^7^. Nevertheless, Kwee et al.^8^ found that biologics or non-biologics systemic therapy did not increase the incidence of SARS-CoV-2 infection. COVID-19 vaccine is an important tool against SARS-CoV-2 infection, with protection effectiveness as high as 95%^9^, but due to breakthrough infection with variants such as Omicron, vaccination with one or more doses only provide 24.7% of effective protection^10^. Regarding psoriasis patients treated with immunosuppressive medications, immunogenicity of the vaccine was impaired and antibody titers decreased^11^. Therefore, real-world studies are required to confirm the effectiveness of vaccine in psoriasis patients confronted with Omicron.

COVID-19 is characterized by excessive host immune response, a cytokine storm due to overproduction of various pro-inflammatory factors such as interleukins (IL) and tumor necrosis factor (TNF), etc^12^, which are common targets for inflammatory diseases such as psoriasis^13^, systematic treatment for these diseases may play a role in COVID-19. Since infection was a trigger factor for psoriasis^14^, there have been case reports about exacerbation or new onset of psoriasis due to SARS-CoV-2 infection^15,16^, but research with large sample sizes and analysis of associated risk factors are lacking.

This study aimed to investigate the prevalence and associated factors of SARS-CoV-2 infection in a real-world setting with psoriasis patients in Southwest China, investigate the factors associated with exacerbation of psoriasis due to SARS-CoV-2 infection, and explore whether psoriasis treatment modalities and vaccination had an impact on COVID-19 to address the above paradoxical issues.

## Methods

### Study design

We conducted a cross-sectional survey in psoriasis patients from three hospital’s dermatology departments of Southwest China through online questionnaire. Patients visiting dermatology clinic with a definitive diagnosis of psoriasis were recruited. Informed consents were collected before conducting the survey. Through the Wen-Juan-Xing platform (Changsha Ranxing Information Technology Co., Ltd., Hunan, China), the online survey was distributed and completed. The questionnaire was conducted between February 2023 and March 2023. All the participants could submit the questionnaire only once. Fully replied questionnaire was considered valid. This study was approved by the ethics committee of The First Affiliated Hospital of Chongqing Medical University, Chongqing, China (Ref no: K2023-080).

### Questionnaires

The questionnaire included information about demographic and clinical characteristics of psoriasis, SARS-CoV-2 infection and prognosis, protection methods against Covid-19. The severity of psoriasis was self-assessed according to the area of skin lesions or body surface area (BSA), and BSA ≤ 3% was classified as mild psoriasis, BSA > 3% was moderate-to-severe psoriasis. According to China SARS-CoV-2 Infection Treatment Protocol (Trial 10th Edition)^17^, the followings were definite as SARS-CoV-2 infection: positive rapid antigen detection (RAD) test or positive polymerase chain reaction (PCR) test with or without COVID-19 symptoms; participants with COVID-19 symptoms and close contact history with confirmed cases, but did not undergo RAD test or PCR test. Denial of SARS-CoV-2 infection: negative PCR test or RAD test with or without symptoms related to respiratory infection. And other undefined cases. At present, there is no consensus on the definition of COVID-19 sequelae, according to the transition from strict quarantine policy to reopening at the specific period in China, we defined COVID-19 sequelae as persistent COVID-19 symptoms for more than four weeks in this study^18^.

### Sample size

According to the estimated infection rate of SARS-CoV-2 in Guangzhou^19^, we presumed that 80% of the patients had been infected with SARS-CoV-2, and about 18 variables were used to explore the risk factors related to SARS-CoV-2 infection. A sample size of 10 times the number of variables was required in the multivariate regression analysis^20^, so 180 participants infected with SARS-CoV-2 were needed. Considering the actual infection rate may exceed 80%, so the minimum sample size was 180/0.8 = 225^21^.

### Statistical analysis

Continuous variables with normal distribution were expressed as mean ± standard deviation (SD). Student T-test were applied to evaluate the difference between groups. If the continuous variables were not subject to normal distribution, the median (interquartile range) was used, and the difference of two groups were evaluated by Mann–Whitney U test. Categorical variables expressed as counts (percentage), compared using Pearson's chi-square test or Fisher's exact test. For multiple comparisons of chi-square test, pairwise chi-square test was performed by Bonferroni correction for P-value. Binary multivariate logistic regression was used to investigate the factors related to SARS-CoV-2 infection and the factors related to the exacerbation of psoriasis. Adjusted odds ratio and 95% confidence interval were used to express the effect sizes. All data were analyzed with SPSS 26 (IBM, SPSS Statistics 26) and Graphpad Prism 8 (GraphPad Software Inc., USA). *P*-value ≤ 0.05 was considered statistically significant.

### Ethical approval

This study was approved by the ethics committee of The First Affiliated Hospital of Chongqing Medical University, Chongqing, China (Ref no: K2023-080) and the study process was conducted in accordance with the committee's requirements. The research process complied with the Declaration of Helsinki. Informed consents were collected before conducting the survey.

## Results

### Baseline features

613 valid questionnaires were collected after removing ones with repeated filling and missing information. The mean values of age and BMI was 43.0 years, 24.1 kg/m^2^, 65.1% were male, 400(65.2%) had full-time or part-time job, 263(42.9%) were undergraduate or above, 404(65.9%) had unhealthy lifestyle habits. Psoriasis vulgaris was the majority type (84.2%, n = 516), and patients with moderate-to-severe psoriasis accounted for 78.0% (n = 478). 63.8% of participants (n = 391) used biologics (Table 1). In case of prophylaxis of COVID-19, 508(82.9%) had routine protection. 568(92.7%) received vaccination, few parts of participants (13.5%, n = 83) had undergone deterioration of psoriasis following COVID-19 vaccination. Among the vaccinated group (n = 568), 274(48.2%) received booster dose vaccination. 516(84.2%) were infected with SARS-CoV-2, among them, the most common symptoms were fever (71.7%, n = 370), cough (56.4%, n = 291), and myalgia (41.7%, n = 215). Most participants (70.7%, n = 365) had COVID-19 symptoms for 7 days or less. 30 (5.8%) were hospitalized or required clinical treatment, 121 (23.4%) discontinued psoriasis treatment and 122(23.6%) had exacerbation of psoriasis due to SARS-CoV-2 infection, 136(26.4%) suffered from COVID-19 sequelae. Fatigue (13.8%, n = 71) and cough (7.2%, n = 37) were the most common symptoms of COVID-19 sequela (Table s1).

**Table 1:** Baseline demographics and clinical characteristics of psoriasis participants.

Characteristics	Total (n = 613)
Age (years), median (IQR)	43.0 (32.0, 56.0)
Male	399 (65.1)
BMI (kg/m^2^), median (IQR)	24.1 (21.8, 26.2)
Working status	
Not working*	180 (29.4)
Full-time/part-time	400 (65.2)
Student	33 (5.4)
Education	
Middle school or below	189 (30.8)
High school	161 (26.3)
College or above	263 (42.9)
Unhealthy lifestyle habit*	
Yes	404 (65.9)
No	209 (34.1)
Subtype of psoriasis	
Psoriasis vulgaris	516 (84.2)
Psoriatic arthritis	81 (13.2)
Pustular psoriasis	7 (1.1)
Erythrodermic psoriasis	9 (1.5)
Course of psoriasis (years)	
≤ 10	294 (48.0)
> 10	319 (52.0)
Severity of psoriasis*	
Mild	135 (22.0)
Moderate to severe	478 (78.0)
Psoriasis treatment	
Oral systemic treatment	86 (14.0)
TCM*	42 (48.8) [of 86]
TYK2*	6 (7.0) [of 86]
Cyclosporin	3 (3.5) [of 86]
Acitretin	26 (30.2) [of 86]
Methotrexate	8 (9.3) [of 86]
Glucocorticosteroid	3 (3.5) [of 86]
Biological treatment	391 (63.8)
Anti TNF-α	31 (8.0) [of 391]
Anti IL-12/23	79 (20.2) [of 391]
Anti IL-23	33 (8.4) [of 391]
Anti IL-17	248 (63.4) [of 391]
Biologics used over 6 months	
Yes	280 (71.6) [of 391]
No	53 (13.6) [of 391]
Unclear	58 (14.8) [of 391]
Non-systemic treatment	97 (15.8)
Not receiving treatment	39 (6.4)
Nail impairment	
Yes	293 (47.8)
No	320 (52.2)
Factors exacerbate psoriasis	
Mental stress	45 (7.3)
Infectious factor	77 (12.6)

Values are presented as n (%) unless stated otherwise. BMI, body mass index; IQR, interquartile range. Unhealthy lifestyle habit*: include smoking, alcohol consumption, bad diet, unlike exercising, poor sleep quality and others. Not working: include retired, unemployed, jobless. Severity of psoriasis*: mild, BSA (body surface area) ≤ 3%; moderate to severe, BSA > 3%. TCM*: Traditional Chinese Medicine; TYK2*: TYK2, tyrosine kinase 2; TNF-α, tumor necrosis factor alpha; IL-12/23, interleukin-12 and 23; IL-23, interleukin-23; IL-17, interleukin-17.

### Psoriasis patients with and without SARS-CoV-2 infection

**Figure 1:**
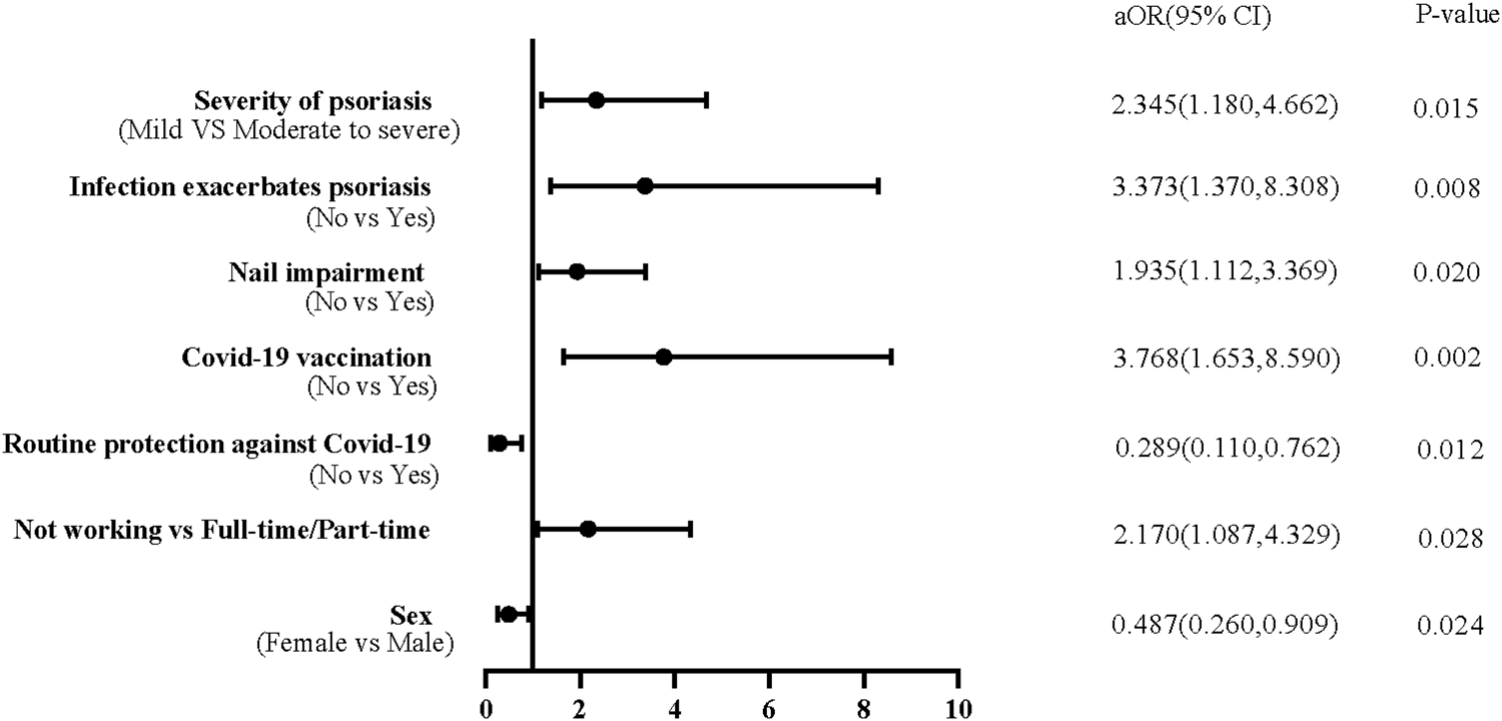
Forest plot for the multivariate regression analysis. In this figure, the position of the circles indicates the adjusted odds ratio (aOR) for each significant factor, and the horizontal lines indicate the 95% CI associated with that value. Y coordinate of the corresponding calibration represents the significant factors associated with SARS-CoV-2 infection. *P*-value of 0.05 or less are considered statistically significant.

Significant differences of features between individuals with and without SARS-CoV-2 infection history were working status, severity of psoriasis, impaired nail, exacerbated psoriasis related to mental stress and infection, COVID-19 vaccination, and routine protection against COVID-19 (Table 2, s2, s3). The proportion of routine protection was higher among unvaccinated participants than vaccinated participants (93.3% vs 80.3%, *p* = 0.031) (Table s4).Adjusted logistic regression analysis was adopted to investigate the factors associated with SARS-CoV-2 infection, it turned out male (aOR = 0.487; 95% CI 0.260–0.909), routine protection (aOR = 0.289; 95% CI 0.110–0.762), vaccination (aOR = 3.768; 95% CI 1.653–8.590), infection exacerbated psoriasis (aOR = 3.373; 95% CI 1.370–8.308), patients with moderate-to-severe psoriasis (aOR = 2.345; 95% CI 1.180–4.662) were associated factors for SARS-CoV-2 infection, patients with full-time or part-time job were more likely be infected with SARS-CoV-2 than those who did not (aOR = 2.170; 95% CI 1.087–4.329), results shown in Table 3 and Fig. 1.

**Table 2:** Clinical characteristics of psoriasis Patients with clear SARS-CoV-2 infection status.

Characteristic	SARS-CoV-2 infection (n = 516)	SARS-CoV-2 non-infection (n = 71)	*P*-value
Subtype of psoriasis			
Psoriasis vulgaris	438 (84.9)	59 (83.1)	0.695
Other subtypes of psoriasis*	78 (15.1)	12 (16.9)	
Course of psoriasis, (years)			
≤ 10	240 (46.5)	38 (53.5)	0.267
> 10	276 (54.5)	33 (46.5)	
Severity of psoriasis*			
Mild	105 (20.3)	22 (31.0)	0.041
Moderate to severe	411 (79.7)	49 (69.0)	
Nail impairment			
Yes	254 (49.2)	26 (36.6)	0.046
No	262(50.8)	45 (63.4)	
Unhealthy lifestyle habits*			
Yes	334 (64.7)	48 (67.6)	0.634
No	182 (35.3)	23 (32.4)	
Mental stress exacerbates psoriasis			
Yes	43 (8.3)	1 (1.4)	0.038
No	473 (91.7)	70 (98.6)	
Infection exacerbates psoriasis			
Yes	130 (25.2)	6 (8.5)	0.002
No	386 (74.8)	65 (91.5)	
Psoriasis treatment			
Oral systemic treatment	71 (13.8)	8 (11.3)	0.933
Biological treatment	330 (63.9)	47 (66.2)	
Non-systemic treatment	82 (15.9)	12 (16.9)	
Not receiving treatment	33 (6.4)	4 (5.6)	
Biologics			
Anti TNF-α	23 (4.5)	6 (8.5)	0.129
Anti IL-12/23	65 (12.6)	13 (18.3)	
Anti IL-23	29 (5.6)	1 (1.4)	
Anti IL-17	213 (41.3)	27 (38.0)	
Oral systemic treatment			
TCM*	36 (7.0)	3 (4.2)	0.306
TYK2*	3 (0.6)	2 (2.8)	
Cyclosporin	2 (0.4)	0 (0.0)	
Acitretin	21 (4.1)	3 (4.2)	
Methotrexate	8 (1.6)	0 (0.0)	
Glucocorticosteroid	4 (0.8)	0 (0.0)	

Values are presented as n (%) unless stated otherwise. Other subtypes of psoriasis*: include Psoriasis Arthritis, Pustular Psoriasis, Erythrodermic Psoriasis. Severity of psoriasis*: mild, BSA (body surface area) ≤ 3%; moderate to severe, BSA > 3%. Unhealthy lifestyle habit*: include smoking, alcohol consumption, bad diet, unlike exercising, poor sleep quality and others. TCM*: Traditional Chinese Medicine; TYK2*: TYK2, tyrosine kinase 2; TNF-α, tumor necrosis factor alpha; IL-12/23, interleukin-12 and 23; IL-23, interleukin-23; IL-17, interleukin-17.

**Table 3:** Logistic regression analysis: factors associated with SARS-CoV-2 infection.

Factors	Unadjusted Model (Univariable analysis)		Adjusted Model (Multivariable analysis)	
	OR (95% CI)	*P*-value	aOR (95% CI)	*P*^a^-value
Age	0.987 (0.970, 1.003)	0.105	0.993 (0.971, 1.016)	0.564
BMI	0.966 (0.907, 1.029)	0.279	0.967 (0.904, 1.033)	0.319
Sex (Female vs Male)	0.573 (0.326, 1.007)	0.053	0.487 (0.260, 0.909)	0.024
Working Status				
Not working	Ref		Ref	
Full-time/part-time	1.878 (1.112, 3.170)	0.018	2.170 (1.087, 4.329)	0.028
Student	1.088 (0.387, 3.059)	0.874	1.151 (0.265, 4.996)	0.851
Routine protection against COVID-19(No vs Yes)	0.315 (0.124, 0.803)	0.015	0.289 (0.110, 0.762)	0.012
COVID-19 Vaccination (No vs Yes)	2.868 (1.371, 6.002)	0.005	3.768 (1.653, 8.590)	0.002
Nail impairment (No vs Yes)	1.678 (1.005, 2.802)	0.048	1.935 (1.112, 3.369)	0.020
Infection exacerbates psoriasis (No vs Yes)	3.649 (1.545, 8.618)	0.003	3.373 (1.370, 8.308)	0.008
Mental stress exacerbates psoriasis (No vs Yes)	6.364 (0.863, 46.948)	0.070	5.075 (0.643, 40.028)	0.123
Psoriasis treatment				
Oral systemic treatment	Ref		Ref	
Biological treatment	0.791 (0.358, 1.747)	0.562	0.630 (0.253, 1.569)	0.321
Non-systemic treatment	0.770 (0.298, 1.990)	0.589	0.721 (0.259, 2.006)	0.531
Not receiving treatment	0.930 (0.261, 3.308)	0.910	0.651 (0.166, 2.557)	0.539
Severity of psoriasis (Mild VS Moderate to severe)	1.757 (1.017, 3.036)	0.043	2.345 (1.180, 4.662)	0.015

Adjustment variables: age, BMI, sex, working status, Routine protection against COVID-19, COVID-19 vaccination, nail impairment, infection exacerbates psoriasis, mental stress exacerbates psoriasis, psoriasis treatment, severity of psoriasis. OR, odds ratio; aOR, adjusted odds ratio; CI, confidence interval.

### Exacerbation of psoriasis due to SARS-CoV-2 infection

**Figure 2:**
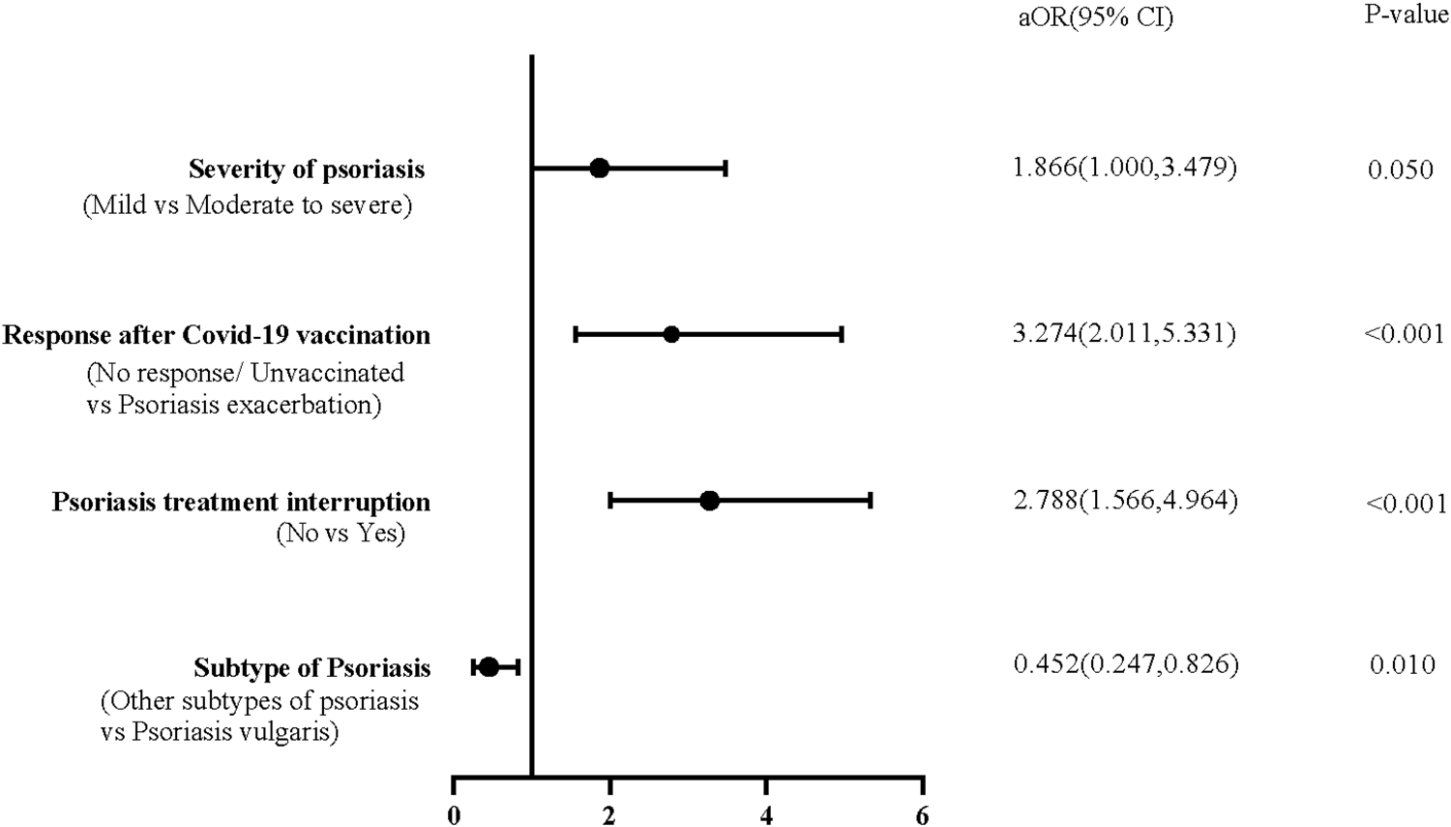
Forest plot for the multivariate regression analysis. In this figure, the position of the circles indicates the adjusted odds ratio (aOR) for each significant factor, and the horizontal lines indicate the 95% CI associated with that value. Y coordinate of the corresponding calibration represents the significant factors associated with psoriasis exacerbation. *P*-value of 0.05 or less are considered statistically significant.

In univariate analysis on correlation of psoriasis exacerbation and SARS-CoV-2 infection, features significantly different between the groups were age, sex, working status, subtype and duration of psoriasis, mental stress exacerbates of psoriasis, treatment of psoriasis, biologics treatment, psoriasis treatment interrupted when COVID-19 infection, exacerbation of psoriasis following COVID-19 vaccination (Table 4, s5, s6). Subsequently, we used adjusted logistic regression analysis, the results were shown in Table 5 and Fig. 2. Compared with other subtypes of psoriasis, psoriasis vulgaris was less likely to be aggregated (aOR = 0.452; 95% CI 0.247–0.826). Patients with discontinuation of anti-psoriasis treatment, flaring-up of psoriasis following COVID-19 vaccination and moderate-to-severe psoriasis were in risk of exacerbation of psoriasis under SARS-CoV-2 infection.

**Table 4:** COVID-19 vaccination characteristics of psoriasis exacerbation due to SARS-CoV-2 infection.

Characteristics	Exacerbation of psoriasis (n = 122)	Non-exacerbation of psoriasis (n = 394)	*P*-value
COVID-19 vaccination			
Yes	9 (7.4)	22 (5.6)	0.466
No	113 (92.6)	372 (94.4)	
Dose of COVID-19 vaccination			
2-dose	62 (50.8)	221 (56.1)	0.597
3-dose	43 (35.2)	136 (34.5)	
COVID-19 booster dose vaccination			
Yes	57 (46.7)	172 (43.7)	0.433
No	56 (45.9)	200 (50.8)	
Exacerbation of psoriasis following COVID-19 vaccination			
Yes	34 (27.9)	40 (10.2)	< 0.001
No and unvaccinated	88 (72.1)	354 (89.8)	

Values are presented as n (%) unless stated otherwise.

**Table 5:** Logistic regression analysis: factors associated with exacerbation of psoriasis.

Factors	Unadjusted model (Univariable analysis)		Adjusted model (Multivariable analysis)	
	OR (95% CI)	*P*-value	aOR (95% CI)	*P*^a^-value
Age	0.980 (0.966, 0.994)	0.006	0.985 (0.966, 1.005)	0.146
BMI	0.965 (0.910, 1.023)	0.232	0.997 (0.937, 1.061)	0.921
Sex (female vs male)	0.621 (0.411, 0.938)	0.024	0.876 (0.541, 1.416)	0.588
Working status				
Not working	Ref		Ref	
Full-time/Part-time	0.850 (0.537, 1.346)	0.489	0.842 (0.464, 1.528)	0.572
Student	2.892 (1.242, 6.736)	0.014	2.602 (0.812, 8.336)	0.107
COVID-19 Vaccination (No vs Yes)	0.743 (0.332, 1.658)	0.468	0.610 (0.251, 1.484)	0.276
Other subtypes of Psoriasis vs Psoriasis vulgaris	0.523 (0.311, 0.879)	0.014	0.452 (0.247, 0.826)	0.010
Course of psoriasis (years)(≤ 10 vs > 10)	0.643 (0.427, 0.967)	0.034	0.828 (0.514, 1.334)	0.438
Mental stress exacerbates psoriasis (No vs Yes)	2.292 (1.198, 4.384)	0.012	1.444 (0.676, 3.084)	0.343
Psoriasis Treatment interruption (No vs Yes)	3.159 (2.029, 4.920)	< 0.001	3.274 (2.011, 5.331)	< 0.001
Response after COVID-19 vaccination (No response/Unvaccinated vs Psoriasis exacerbation)	3.419 (2.046, 5.713)	< 0.001	2.788 (1.566, 4.964)	< 0.001
Psoriasis treatment				
Oral systemic treatment	Ref		Ref	
Biological treatment	0.502 (0.285, 0.885)	0.017	0.562 (0.292, 1.083)	0.085
Non-systemic treatment	0.864 (0.434, 1.718)	0.676	0.958 (0.444, 2.064)	0.912
Not receiving treatment	1.043 (0.434, 2.511)	0.924	1.071 (0.404, 2.842)	0.890
Severity of psoriasis (Mild VS Moderate to severe)	1.131 (0.676, 1.893)	0.639	1.866 (1.000, 3.479)	0.050

Adjustment variables: age, BMI, sex, working status, COVID-19 vaccination, types of psoriasis, course of psoriasis, mental stress exacerbates psoriasis, psoriasis treatment interruption, response after COVID-19 vaccination, psoriasis treatment, severity of psoriasis. OR, odds ratio; aOR, adjusted odds ratio; CI, confidence interval.

### SARS-CoV-2 infection in vaccinated psoriasis patients

In our cohort, the types of vaccines were mostly inactivated (2-dose) and recombinant protein (3-dose) vaccines. Vaccination was not an interfering factor in the course of COVID-19, COVID-19 hospitalization and the occurrence of COVID-19 sequelae (*p* > 0.05). Patients who received 2-dose of vaccine had lower probabilities of COVID-19 sequelae than those who received 3-dose (20.1% vs 33.5%, *p* = 0.001). Patients did not receive booster dose vaccine were more likely to have COVID-19 sequelae (*p* = 0.027) (Table s7–s9).

### SARS-CoV-2 infection in psoriasis patients with psoriasis treatment

Among patients treated with all kinds of modalities and untreated patients, no difference in the duration of COVID-19, occurrence of sequelae or COVID-19 hospitalization (*P* > 0.05). In contrast, for biologics, different probability of COVID-19 sequelae occurrence correlated with certain biologics (*p* = 0.012), by pairwise comparisons, we concluded that the IL-23 inhibitor generated the lowest rate of COVID-19 sequelae compared to the other three biologics (*p* < 0.0083), which also had the advantage of preventing the deterioration of psoriasis (Table s10–s12).

## Discussion

Since the end of the dynamic zero-COVID policy, the incidence of SARS-CoV-2 infection in mainland China was skyrocketing during a short period. A large sample survey^19^ predicted that SARS-CoV-2 infection in mainland China would reach 80.7% on the 30th day after the shift of epidemic prevention policy, another regional study^22^ predicted the prevalence was 88.5%. Likewise, our study came to a close prediction, which suggests that Chinese psoriasis patients have a comparable incidence of SARS-CoV-2 infection with the general population.

Previous study^23^ revealed risk factors for SARS-CoV-2 infection in the general population include advanced age, male gender, etc. In our study, females were more susceptible to SARS-CoV-2 infection than males, however, it was still debatable whether gender affected SARS-CoV-2 infection^24^. SARS-CoV-2 enters the body through angiotensin-converting enzyme 2 (ACE2), and expression level of ACE2 is higher in male than female, theoretically, male should be more susceptible to SARS-CoV-2 infection^25^, whether there is modulation in ACE2 expression of both genders in patients with psoriasis? Further researches are needed to determine whether males with psoriasis are more susceptible to SARS-CoV-2 infection.

Patients with impaired nail and moderate-to-severe psoriasis were more likely to be infected, since impaired nail often indicated severe cases of psoriasis^26^, suggesting that psoriasis severity is a predictor of infection risk^7^. Patients with severe psoriasis express high level of interferon (IFN)^27^, which in term promote the expression of ACE2^28^, therefore have a higher risk of contracting SARS-CoV-2. Infection in general was an environmental factor in the exacerbation or triggering of psoriasis^14^, and patients who experienced exacerbation of psoriasis by infection were more likely to be contracted by SARS-CoV-2.

Our analysis came up with an interesting finding. We noticed that COVID-19 vaccine did not offer valid protection in Chinese psoriasis patients, instead it was associated with the occurrence of SARS-CoV-2 infection for possible reasons: (1) Breakthrough infection with the Omicron variant. Omicron was endemic in mainland China around the end of the dynamic zero-COVID policy^29^, it exhibited higher transmissibility and lower susceptibility to neutralizing antibodies induced by vaccine compared to the original strain^3^. At the same time, immunity stimulated by vaccine was waning over time^30^; (2) Lack of routine protection against COVID-19. In our cohort, ratio of routine protection was lower in vaccinated psoriasis patients than in unvaccinated patients. Our findings and previous studies^31^ have reached consensus routine protection against COVID-19 was a protective factor against SARS-CoV-2 infection. Therefore, we suggest that routine protection should not be neglected even under vaccinated status; (3) Low naturally acquired immunity. A study^32^ reported that naturally acquired immunity played more crucial roles in preventing infection than vaccination, while under the previous dynamic zero-COVID policy, the general infection rate in China was at a low level, and herd immunity in Chinese population was far from robust compared with other countries^33^; (4) Whether psoriasis patients were suitable for COVID-19 vaccination remains controversial. No unified conclusion on whether COVID-19 vaccine in psoriasis patients could stimulate valid specific immunity, especially in patients receiving long-term immunosuppressive therapy with impaired humoral response to the vaccine^34^, who constituted the majority of our cohort.

The pathogenesis of psoriasis has not been fully elucidated. Until now, the general research supports that the IL-23/T-helper cell type 17 (Th17) axis dominates the inflammation activation process, and various cytokines such as IL-17, TNF, and IL-12 are involved^13^, which intersects with the pro-inflammatory factors induced by SARS-CoV-2^35^. Aram et al.^15^ summarized case reports of psoriasis exacerbation due to SARS-CoV-2 infection, but did not investigate factors associated with exacerbation of psoriasis. In our study, patients with moderate-to-severe psoriasis were more prone to undergo exacerbation, which might due to the fact that they bear a stronger basic inflammatory process^27^, and when SARS-CoV-2 superimposed, it further ignited the inflammation process. Discontinuation of treatment can lead to recurrence or exacerbation of psoriasis^36^. Tissue-resident memory (TRM) T cells are involved in the recurrence of psoriasis after treatment discontinuation. TRM cells can be divided into CD4^+^ and CD8^+^ subsets, which respectively promote the production of IL-22 and IL-17A, contribute to an inflammatory environment in localized tissues^37^. SARS-CoV-2 infection also caused exacerbation of psoriasis in patients who had exacerbated after previous COVID-19 vaccination, a review^38^ even reported new onset of psoriasis after COVID-19 vaccination. The mechanism might be vaccination activating of both CD4^+^ and CD8^+^ T cells, increasing levels of INF-γ, TNF-α, IL-2 and IL-12^39^.

Whether psoriasis patients receiving immunosuppressive therapy were at higher risk of SARS-CoV-2 infection and prone to COVID-19 adverse events remains unsolved. A multicenter study^40^ based on 11,466 patients found an increased risk of infection in psoriasis patients under TNF inhibitor therapy. However, Liu et al.^41^ reported no difference in SARS-CoV-2 infection, COVID-19 hospitalization in psoriasis patients using IL-17 inhibitor compared to non-biologic agents. In our study, psoriasis treatment modalities were not associated with SARS-CoV-2 infection and COVID-19 hospitalization. Therefore, discontinuing psoriasis treatment is not recommended in the setting of SARS-CoV-2 infection except in the case of a severe COVID-19 event, and active treatment of psoriasis is beneficial in preventing SARS-CoV-2 infection and preventing exacerbation of psoriasis due to SARS-CoV-2 infection.

Although our results indicated that COVID-19 vaccination (including booster dose) in little help in prevention of SARS-CoV-2 infection or reduction of COVID-19 hospitalization. Booster dose vaccination did reduce the incidence of COVID-19 sequelae, the result was similar to a meta-analysis^42^ by Gao. Therefore, booster dose vaccination is recommended.

This is the pioneer study concerning impact of SARS-CoV-2 infection on psoriasis after the end of dynamic zero-COVID policy in mainland China, in real-world examining effectiveness of COVID-19 vaccine in psoriasis population with low naturally acquired immunity, and answering the linkage between psoriasis treatment and COVID-19-related events. We also explored the risk factors for exacerbation of psoriasis due to SARS-CoV-2 infection. Because of the high variability of the SARS-CoV-2, another large-scale impact on psoriasis patients is still possible, the findings of our work may contribute to the exploration of psoriasis and SARS-CoV-2 related issues.

There are some limitations of this study. First, regarding the screening of participants, in order to best simulate the situation of SARS-CoV-2 infection at that time, and the limitation of PCR testing and lack of antigen detection kits, we lowered the criteria for confirming SARS-CoV-2 infection to screen as many SARS-CoV-2 infection as possible, which may lead to an increase in the false positive rate. Secondly, despite the fact that the research included three hospitals and some patients were spread throughout many provinces in Southwest China, the majority of them were concentrated in one area. Because some of the patients were elderly and not sure what kind of vaccine they had received, so we did not explore the association between specific vaccine subtypes and SARS-CoV-2 infection. Furthermore, the authenticity of the data may be limited by questionnaires, as psoriasis severity was based on patients' own assessment of BSA. Except that, this survey was completed online, and there might be a selective bias due to the low participation rate of geriatric and pediatric patients.

## Conclusion

We found that SARS-CoV-2 infection was associated with COVID-19 vaccination, while booster dose vaccination assisted in lowering the incidence of COVID-19 sequelae. Other major SARS-CoV-2 infection risk factors were female gender, employed individuals, lack of routine protection, severe cases of psoriasis. Associated factors for psoriasis exacerbation were subtypes of psoriasis, response following COVID-19 vaccination, severity of psoriasis, discontinuation of psoriasis treatment. Psoriasis treatment was unrelated to SARS-CoV-2 infection and COVID-19 hospitalization, biologics proved safety during SARS-COV-2 infection.

### Supplementary Information

Supplementary Tables.

## Data Availability

The data that support the findings of this study are available from the corresponding author, upon reasonable request.
