# Supplementary material for: Prevalence, outcomes and associated factors of SARS-CoV-2 infection in psoriasis patients of Southwest China: a cross-sectional survey
Source: Sci Rep. 2024 Mar 15;14:6331. doi: 10.1038/s41598-024-54424-y (PMC10943245; doi:10.1038/s41598-024-54424-y)
Supplement: Supplementary file 1 — Supplementary Tables. [file 41598_2024_54424_MOESM1_ESM.docx]

Supplementary data:

Table s1. SARS-CoV-2 infection, protection against COVID-19 and vaccination characteristics of psoriasis participants

| Characteristics | Total(n=613) |
| --- | --- |
| **COVID-19 vaccination** |  |
| Yes | 568(92.7) |
| 2-dose | 340(59.9) [of 568] |
| 3-dose | 203(35.7) [of 568] |
| Unclear* | 25(4.4) [of 568] |
| No | 45(7.3) |
| **COVID-19 booster dose vaccination** |  |
| Yes | 274(48.2) [of 568] |
| No | 294(51.8) |
| **Exacerbation of psoriasis following COVID-19 vaccination** |  |
| Yes | 83(13.5) |
| No and unvaccinated | 530(86.5) |
| **Routine protection against COVID-19*** |  |
| Yes | 508(82.9) |
| No | 105(17.1) |
| **Whether infected with SARS-CoV-2** |  |
| Yes | 516(84.2) |
| No | 71(11.6) |
| Unclear | 26(4.2) |
| **COVID-19 symptoms** |  |
| Asymptomatic infection | 15(3.0) [of 516] |
| Fever | 370(71.7) |
| Cough | 291(56.4) |
| Rhinitis | 52(10.1) |
| Dyspnea | 21(4.1) |
| Myalgia | 215(41.7) |
| Headache | 126(24.4) |
| Loss of taste | 21(4.1) |
| Loss of smell | 72(14.0) |
| Others | 58(11.2) |
| **Course of COVID-19** |  |
| ≤7 | 365(70.7) [of 516] |
| >7 | 151(29.3) |
| **Hospitalization or clinic treatment of COVID-19** |  |
| Yes | 30(5.8) [of 516] |
| No | 486(94.2) |
| **Psoriasis treatment interrupted when SARS-CoV-2 infection** |  |
| Yes | 121(23.4) [of 516] |
| No | 395(76.6) |
| **Exacerbation of psoriasis because of SARS-CoV-2 infection** |  |
| Yes | 122(23.6) [of 516] |
| No | 394(76.4) |
| **COVID-19 sequelae** |  |
| Yes | 136(26.4) [of 516] |
| Fatigue | 71(13.8) |
| Cough | 37(7.2) |
| Attention disorder | 22(4.3) |
| Dyspnea | 16(3.1) |
| CT lung abnormalities | 8(1.6) |
| Myalgia | 21(4.1) |
| Eye problems | 15(2.9) |
| Psychiatric illness | 11(2.1) |
| Others | 20(3.9) |
| No | 380(73.6) |

Values are presented as n (%) unless stated otherwise. Unclear*: non-full dose vaccination or unclear vaccination dose. Routine protection against COVID-19*: wash hands after and before touching object, wear a mask in the public, maintain good respiratory hygiene and others.

Table s2. Demographic characteristics of psoriasis Patients with clear SARS-CoV-2 infection status:

| Characteristic | SARS-CoV-2 infection (n=516) | SARS-CoV-2 non-infection(n=71) | P-value |
| --- | --- | --- | --- |
| **Age (years), median (IQR)** | 42(32,55) | 45(35,60) | 0.093 |
| **Male** | 324(62.8) | 53(74.6) | 0.051 |
| **BMI (kg/m^2^)，mean ± SD** | 24.2±3.6 | 24.7±4.4 | 0.352 |
| **Working status**  Not working*  Full-time/ Part-time  Student | 144(27.9)  345(66.9)  27(5.2) | 29(40.8)  37(52.1)  5(7.1) | 0.050 |
| **Education**  Middle school or below  High school  College or above | 154(29.8)  137(26.6)  225(43.6) | 26(36.6)  18(25.4)  27(38.0) | 0.492 |

Values are presented as n (%) unless stated otherwise. BMI, body mass index; IQR, interquartile range. Not working: include retired, unemployed, jobless.

Table s3. COVID-19 vaccination and routine protection against COVID-19 of psoriasis Patients with clear SARS-CoV-2 infection status:

| Characteristic | SARS-CoV-2 infection (n=516) | | SARS-CoV-2 non-infection (n=71) | | P-value | |
| --- | --- | --- | --- | --- | --- | --- |
| **Routine protection against COVID-19**  Yes  No | 416(80.6)  100(19.4) | 65(91.5)  6(8.5) | | 0.025 | |  |
| **COVID-19 vaccination**  Yes  No | 485(94.0)  31(6.0) | 60(84.5)  11(15.5) | | 0.004 | |  |
| **Dose of COVID-19**  **vaccination**  2-dose  3-dose | 283(54.8)  139(26.9) | 39(54.9)  20(28.2) | | 0.471 | |  |
| **COVID-19 booster dose vaccination**  Yes  No | 229(44.4)  256(49.6) | 28(39.4)  32(45.1) | | 0.936 | |  |
| **Exacerbation of psoriasis following COVID-19 vaccination**  Yes  No and unvaccinated | 74(14.3)  442(85.7) | 8(11.3)  63(78.7) | | 0.586 | |  |

Values are presented as n (%) unless stated otherwise.

Table s4. Routine protection against COVID-19 in patients vaccinated and unvaccinated:

| Characteristic | Vaccinated (n=568) | Unvaccinated (45) | P-value |
| --- | --- | --- | --- |
| **Routine protection against COVID-19** |  |  |  |
| Yes  No | 456(80.3)  112(19.7) | 42(93.3)  3(6.7) | 0.031 |

Values are presented as n (%) unless stated otherwise.

Table s5. Demographic characteristics of psoriasis exacerbation due to SARS-CoV-2 infection:

| Characteristics | Exacerbation of psoriasis (n=122) | Non-exacerbation of psoriasis (n=394) | P-value |
| --- | --- | --- | --- |
| **Age (years), median (IQR)** | 37.0(30.0,50.0) | 44.0(32.0,56.0) | 0.006 |
| **Male** | 66(54.1) | 258(65.5) | 0.023 |
| **BMI (kg/m2), median (IQR)** | 23.4(21.4,26.3) | 24.2(21.9,26.0) | 0.251 |
| **Working status**  Not working*  Full-time/ Part-time  Student | 35(28.7)  74(60.6)  13(10.7) | 109(27.7)  271(68.7)  14(3.6) | 0.007 |
| **Education**  Middle school or below  High school  College or above | 36(29.5)  30(24.6)  56(45.9) | 118(30.0)  107(27.2)  169(42.8) | 0.806 |

Values are presented as n (%) unless stated otherwise. BMI, body mass index; IQR, interquartile range. Not working: include retired, unemployed, jobless.

Table s6. Clinical characteristics of psoriasis exacerbation due to SARS-CoV-2 infection:

| Characteristics | Exacerbation of psoriasis (n=122) | Non-exacerbation of psoriasis (n=394) | P-value |
| --- | --- | --- | --- |
| **Subtype of psoriasis**  Psoriasis vulgaris  Other subtypes of psoriasis | 95(77.9)  27(22.1) | 343(87.1)  51(12.9) | 0.013 |
| **Course of psoriasis (years)**  ≤10  >10 | 67(54.9)  55(45.1) | 173(43.9)  221(56.1) | 0.033 |
| **Severity of psoriasis***  Mild  Moderate to severe | 23(18.9)  99(81.1) | 82(20.8)  312(79.2) | 0.585 |
| **Nail impairment**  Yes  No | 51(41.8)  71(58.2) | 203(51.5)  191(48.5) | 0.061 |
| **Unhealthy lifestyle habits**  Yes  No | 81(66.4)  41(33.6) | 253(64.2)  141(35.8) | 0.660 |
| **Mental stress exacerbates psoriasis**  Yes  No | 17(13.9)  105(86.1) | 26(6.6)  368(93.4) | 0.010 |
| **Psoriasis treatment**  Oral systemic treatment  Biological treatment  Non-systemic treatment  Not receiving treatment  **Biologics**  Anti TNF- α  Anti IL-12/23  Anti IL-23  Anti IL-17  **Oral systemic treatment**  TCM*  TYK2*  Cyclosporin  Acitretin  Methotrexate  Glucocorticosteroid | 23(18.9)  64(52.4)  24(19.7)  11(9.0)  6(4.9)  16(13.1)  0(0.0)  42(34.4)  10(8.2)  0(0.0)  0(0.0)  10(8.2)  2(1.6)  3(2.5) | 48(12.2)  266(67.5)  58(14.7)  22(5.6)  17(4.3)  49(12.4)  29(7.4)  171(43.4)  26(6.6)  3(0.8)  2(0.5)  11(2.8)  6(1.5)  2(0.5) | 0.024  0.032  0.297 |
| **Psoriasis treatment interrupted when SARS-CoV-2 infection**  Yes  No | 50(41.0)  72(59.0) | 71(18.0)  323(82.0) | <0.001 |

Values are presented as n (%) unless stated otherwise. Other subtypes of psoriasis*: include Psoriasis Arthritis, Pustular Psoriasis, Erythrodermic Psoriasis. Severity of psoriasis*: mild, BSA (body surface area) ≤3%; moderate to severe, BSA>3%. TCM*: Traditional Chinese Medicine; TYK2*: TYK2, tyrosine kinase 2; TNF- α, tumor necrosis factor alpha; IL-12/23, interleukin-12 and 23; IL-23, interleukin-23; IL-17, interleukin-17.

Table s7. Characteristics of SARS-CoV-2 infection in psoriatic patients with COVID-19 vaccination:

| Characteristics | Unvaccinated(n=31) | Vaccinated(n=485) | P-value |
| --- | --- | --- | --- |
| **Course of COVID-19**  ≤7 d  >7 d | 19(61.3)  12(38.7) | 346(71.3)  139(28.7) | 0.233 |
| **COVID-19 sequelae**  Yes  No | 7(22.6)  24(77.4) | 129(26.6)  356(73.4) | 0.623 |

Values are presented as n (%) unless stated otherwise.

Table s8. Characteristics of SARS-CoV-2 infection in psoriasis patients vaccinated against COVID-19.

| Characteristics | vaccinated | | | | | |  |
| --- | --- | --- | --- | --- | --- | --- | --- |
|  | 2-dose(n=283) | 3-dose(n=179) | P-value | Booster dose  unvaccinated(n=256) | Booster dose  vaccinated (n=229) | P-value |  |
| **Course of COVID-19**  ≤7 d  >7 d | 204(72.1)  79(27.9) | 130(72.6)  49(27.4) | 0.899 | 177(69.1)  79(30.9) | 169(73.8)  60(26.2) | 0.257 |  |
| **COVID-19 sequelae**  Yes  No | 57(20.1)  226(79.9) | 60(33.5)  119(66.5) | 0.001 | 79(30.9)  177(69.1) | 50(21.8)  179(78.2) | 0.027 |  |

Values are presented as n (%) unless stated otherwise.

Table s9. COVID-19 vaccination of psoriatic Patients with COVID-19 hospitalization:

| Characteristic | Hospitalization (n=30) | Non-hospitalization (n=486) | P-value |
| --- | --- | --- | --- |
| **COVID-19 vaccination**  Yes  No | 27(90.0)  3(10.0) | 458(94.2)  28(5.8) | 0.343 |
| **Dose of COVID-19 vaccination**  2-dose  3-dose | 16(53.3)  9(30.0) | 267(54.9)  170(35.0) | 0.772 |
| **COVID-19 booster dose vaccination**  Yes  No | 10(33.3)  17(56.7) | 219(45.1)  239(49.2) | 0.276 |
| **Exacerbation of psoriasis following COVID-19 vaccination**  Yes  No and unvaccinated | 8(26.7)  22(73.3) | 66(13.6)  420(86.4) | 0.086 |

Values are presented as n (%) unless stated otherwise.

Table s10. Characteristics of SARS-CoV-2 infection in psoriasis patients with psoriasis treatment:

| Characteristics | Treatment | | | | |
| --- | --- | --- | --- | --- | --- |
|  | Oral systemic  (n=71) | Biological  (n=330) | Non-systemic  (n=82) | Non-treatment  (n=33) | P-value |
| **Course of COVID-19**  ≤7 d  >7 d | 56(78.9)  15(21.1) | 230(69.7)  100(30.) | 53(64.6)  29(35.4) | 26(78.8)  7(21.2) | 0.175 |
| **COVID-19 sequelae**  Yes  No | 15(21.1)  56(78.9) | 97(29.4)  233(70.6) | 16(19.5)  66(80.5) | 8(24.2)  25(75.8) | 0.202 |

Values are presented as n (%) unless stated otherwise.

Table s11. Clinical characteristics of psoriatic Patients with COVID-19 hospitalization:

| Characteristic | Hospitalization (n=30) | Non-hospitalization(n=486) | P-value |
| --- | --- | --- | --- |
| **Subtype of psoriasis**  Psoriasis vulgaris  Other subtypes of psoriasis | 26(86.7)  4(13.3) | 412(84.8)  74(15.2) | 0.779 |
| **Course of psoriasis (years)**  ≤10  >10 | 15(50.0)  15(50.0) | 225(46.3)  261(53.7) | 0.693 |
| **Severity of psoriasis***  Mild  Moderate to severe | 4(13.3)  26(86.7) | 101(20.8)  385(79.2) | 0.325 |
| **Nail impairment**  Yes  No | 15(50.0)  15(50.0) | 239(49.2)  247(50.8) | 0.930 |
| **Unhealthy lifestyle habits**  Yes  No | 18(60.0)  12(40.0) | 316(65.0)  170(35.0) | 0.577 |
| **Mental stress exacerbates psoriasis**  Yes  No | 5(16.7)  25(83.3) | 38(7.8)  448(92.2) | 0.173 |
| **Infection exacerbates psoriasis**  Yes  No | 12(40.0)  18(60.0) | 118(24.3)  368(75.7) | 0.054 |
| **Psoriasis treatment**  Oral systemic treatment  Biological treatment  Non-systemic treatment  Not receiving treatment | 6(20.0)  17(56.7)  7(23.3)  0(0.0) | 65(13.4)  313(64.4)  75(15.4)  33(6.8) | 0.230 |
| Biologics  Anti TNF- α  Anti IL-12/23  Anti IL-23  Anti IL-17 | 2(6.7)  3(10.0)  2(6.7)  10(33.3) | 21(4.3)  62(12.8)  27(5.6)  203(41.8) | 0.825 |
| Biologics used over 6 months  Yes  No  Oral systemic treatment  TCM*  TYK2*  Cyclosporin  Acitretin  Methotrexate  Glucocorticosteroid | 12(40.0)  4(13.3)  4(13.3)  1(3.3)  0(0.0)  1(3.3)  0(3.3)  0(0.0) | 227(46.7)  38(7.8)  32(6.6)  2(0.4)  2(0.4)  20(4.1)  8(1.6)  5(1.04) | 0.423  0.503 |
| **Psoriasis treatment interrupted when**  **SARS-CoV-2 infection**  Yes  No | 10(33.3)  20(66.7) | 111(22.8)  375(77.2) | 0.188 |

Values are presented as n (%) unless stated otherwise. Other subtypes of psoriasis*: include Psoriatic Arthritis, Pustular Psoriasis, Erythrodermic Psoriasis. Severity of psoriasis*: mild, BSA (body surface area) ≤3%; moderate to severe, BSA>3%. TCM*: Traditional Chinese Medicine; TYK2*: TYK2, tyrosine kinase 2; TNF- α, tumor necrosis factor alpha; IL-12/23, interleukin-12 and 23; IL-23, interleukin-23; IL-17, interleukin-17.

Table s12. Characteristics of SARS-CoV-2 infection in psoriatic patients with psoriasis biological treatment:

| Characteristics | Biological treatment | | | | |
| --- | --- | --- | --- | --- | --- |
|  | Anti TNF-α(n=23) | Anti IL-12/23(n=65) | Anti IL-23(n=29) | Anti IL-17(n=213) | P-value |
| **Exacerbation of psoriasis**  Yes  No  **Course of COVID-19**  ≤7 d  >7 d | 6(26.1)  17(73.9)  16(69.6)  7(30.4) | 16(24.6)  49(75.4)  41(63.1)  24(36.9) | 0(0.0)  29(100.0)  22(75.9)  7(24.1) | 42(19.7)  171(80.3)  151(70.9)  62(29.1) | 0.032  0.569 |
| **COVID-19 sequelae**  Yes  No | 8(34.8)  15(65.2) | 23(35.4)  42(64.6) | 1(3.4)  28(96.6) | 65(30.5)  148(69.5) | 0.012 |

Values are presented as n (%) unless stated otherwise.
